# Bis(μ-2-*tert*-butyl­phenyl­imido-1:2κ^2^ *N*:*N*)chlorido-2κ*Cl*-(diethyl ether-1κ*O*)(2η^5^-penta­methyl­cyclo­penta­dien­yl)lithiumtantalum(V)

**DOI:** 10.1107/S1600536811015650

**Published:** 2011-05-07

**Authors:** Jacqueline M. Cole, Michael C. W. Chan, Vernon C. Gibson, Judith A. K. Howard

**Affiliations:** a Cavendish Laboratory, University of Cambridge, J. J. Thomson Avenue, Cambridge CB3 0HE, England; b Department of Biology and Chemistry, City University of Hong Kong, Tat Chee Avenue, Kowloon Tong, Kowloon, Hong Kong; c Department of Chemistry, Imperial College London, Exhibition Road, London SW7 2AZ, England; d Department of Chemistry, University of Durham, South Road, Durham DH1 3LE, England

## Abstract

In the title compound, [LiTa(C_10_H_15_)(C_10_H_13_N)_2_Cl(C_4_H_10_O)], the Ta^V^ atom is coordinated by a η^5^-penta­methyl­cyclo­penta­dienyl (Cp*) ligand, a chloride ion and two *N*-bonded 2-*tert*-butyl­phenyl­imide dianions. With respect to the two N atoms, the chloride ion and the centroid of the Cp* ring, the tantalum coordination geometry is approximately tetra­hedral. The lithium cation is bonded to both the 2-*tert*-butyl­phenyl­imide dianions and also a diethyl ether mol­ecule, in an approximate trigonal planar arrangement. The Ta⋯Li separation is 2.681 (15) Å. In the crystal, a weak C—H⋯Cl inter­action links the mol­ecules. When compared to the 2,6-diisopropyl­phenyl­imide analogue (‘the Wigley derivative’) of the title compound, the two structures are conformationally matched with an overall r.m.s. difference of 0.461Å.

## Related literature

For related work demonstrating the stabilization of unusual imido metal species *via* 2,6-diisopropyl­phenyl substitution, see: Cockcroft *et al.* (1992 ▶); Glueck *et al.* (1991 ▶); Anhaus *et al.* (1990 ▶); Gibson & Poole (1995 ▶); Baldwin *et al.* (1993 ▶). For conformational analysis of structures, see: Weng *et al.* (2008 ▶). For van der Waals contact distances, see: Bondi (1964 ▶). For crystal mounting techniques, see: Kottke & Stalke (1993 ▶).
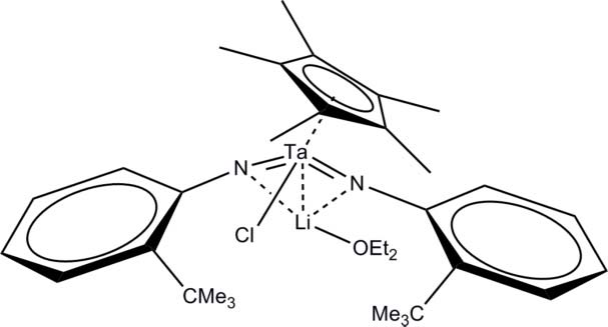


## Experimental

### 

#### Crystal data

[LiTa(C_10_H_15_)(C_10_H_13_N)_2_Cl(C_4_H_10_O)]
*M* *_r_* = 727.11
Orthorhombic, 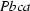

*a* = 19.5365 (12) Å
*b* = 16.3544 (10) Å
*c* = 21.3272 (13) Å
*V* = 6814.2 (7) Å^3^
*Z* = 8
Mo *K*α radiation
μ = 3.33 mm^−1^
*T* = 150 K
0.60 × 0.34 × 0.16 mm

#### Data collection

Siemens SMART CCD diffractometer
Absorption correction: multi-scan (*SADABS*; Siemens, 1995 ▶) *T*_min_ = 0.346, *T*_max_ = 0.666
24495 measured reflections
4848 independent reflections
4792 reflections with *I* > 2σ(*I*)
*R*_int_ = 0.060
θ_max_ = 23.3°

#### Refinement

*R*[*F* ^2^ > 2σ(*F* ^2^)] = 0.053
*wR*(*F* ^2^) = 0.105
*S* = 1.23
4848 reflections
337 parameters
1 restraint
H-atom parameters constrained
Δρ_max_ = 1.29 e Å^−3^
Δρ_min_ = −0.95 e Å^−3^

### 

Data collection: *SMART* (Siemens, 1995 ▶); cell refinement: *SAINT* (Siemens, 1995 ▶); data reduction: *SAINT*; program(s) used to solve structure: *SHELXS86* (Sheldrick, 2008 ▶); program(s) used to refine structure: *SHELXL93* (Sheldrick, 2008 ▶); molecular graphics: *SHELXTL* (Sheldrick, 2008 ▶) and *Mercury* (Macrae *et al.*, 2008 ▶); software used to prepare material for publication: *SHELXL93*.

## Supplementary Material

Crystal structure: contains datablocks I, global. DOI: 10.1107/S1600536811015650/hb5832sup1.cif

Structure factors: contains datablocks I. DOI: 10.1107/S1600536811015650/hb5832Isup2.hkl

Additional supplementary materials:  crystallographic information; 3D view; checkCIF report

## Figures and Tables

**Table 1:** Selected bond lengths (Å)

Ta1—N1	1.842 (6)
Ta1—N2	1.854 (6)
Ta1—Cl1	2.3985 (19)
Li1—N1	2.048 (16)
Li1—N2	2.062 (16)
Li1—O1	1.910 (19)

**Table 2:** Hydrogen-bond geometry (Å, °)

*D*—H⋯*A*	*D*—H	H⋯*A*	*D*⋯*A*	*D*—H⋯*A*
C12—H12*A*⋯Cl1^i^	0.95	2.89	3.593 (8)	132

Symmetry code: (i) 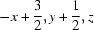.

**Fig. 1.**
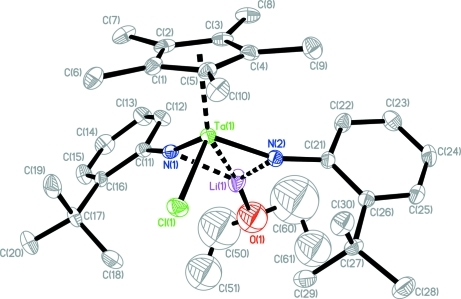
The molecular structure of (I). Displacement parameters are displayed at the 30% probability level. Hydrogen atoms have been omitted for clarity.

**Fig. 2.**
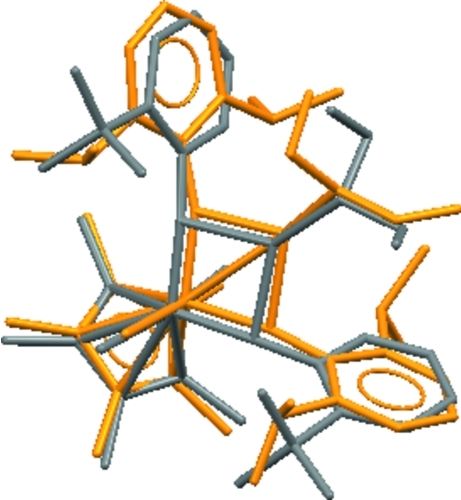
Best fit overlay of molecules (I) and (II).

**Fig. 3.**
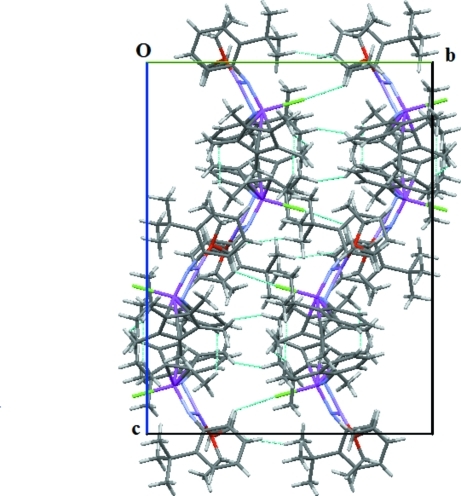
Hydrogen-bonding linking chains of molecules in (I) along the crystallographic y-axis.

## supplementary crystallographic information

### Comment

The bulky 2,6-diisopropylphenyl substituent has been investigated widely in transition metal imido chemistry, and has been shown to stabilize a variety of unusual imido metal species (Cockcroft *et al.*, 1992; Glueck *et al.*, 1991; Anhaus *et al.*, 1990; Gibson & Poole, 1995). The presence of two bulky *ortho* isopropyl substituents undoubtedly plays an important role in this stabilization. Imido aryl substituents containing one bulky substituent in the *ortho* position also offer the possibility for substantial steric protection, not only due to the presence of the large substituent but also as a result of bending at the imido nitrogen. We have thus studied the bis (2 - t-butylphenylimido) chloro (η^5^-pentamethylcyclopentadienyl) tantalum(V) anion (I) with a view to comparing its structure with its previously reported 2,6-diisopropylphenylimido analogue (II) (Baldwin *et al.*, 1993).

The 50% probability thermal ellipsoid plot of the molecular structure of (I) is given in Figure 1. Selected bond distances and angles are given in Table 1. Fractional coordinates and anisotropic displacement parameters are provided in supplementary material.

The overall bond geometry of the title compound is generally similar to its 2,6-diisopropylphenylimido analogue. In particular, the Ta(1)—N(1) and Ta(1)—N(2) distances and Ta(1)—N(1)—C(11) and Ta(1)—N(2)—C(21) angles are comparable [1.844 (6) Å, 1.848 (6) Å, 165.9 (5)° and 161.7 (5)° repectively].

However, several geometrical differences exist between the two compounds as a result of the presence of a more bulky aryl imido substituent in this case.

In the 2,6-diisopropylphenylimido structure, the planes of the arylimido and Cp* rings are approximately parallel to each other to minimize steric repulsion between the respective isopropyl and methyl groups. The situation for the 2 - t-butylphenylimido congener is comparable, except that the single bulky *tert*-butyl substituent on each imido ligand is now positioned in a less congested orientation away from the [µ-Li(OEt_2_)]+ moiety, such that they point in a similar direction to the Ta—Cl vector.

This steric alleviation is shown in Figure 2, which also illustrates the overall conformational difference between the two structures. This structure overlay was generated by matching the following respective atom pairs in each molecule: Ta, N, Li, O, Cp* and phenyl C atoms (Weng *et al.*, 2008). These atoms are conformationally matched with an overall root-mean-square difference of 0.461 Å. The geometric differences between the *tert*-butyl and disopropyl phenyl substituents are emphasized by the visual offset to this conformationally matched molecular fragment. A full list of individual atomic pairwise deviations from a perfect match is given in supplementary information.

The Ta(1)—Li(1) distance of 2.68 (1)Å is slightly longer than in the Wigley derivative, being the only other reported. We presume that this is also consequent upon the greater steric repulsion from the tertiary butyl groups compared to the isopropyl groups of the phenylimido ligands.

The atoms in the OEt_2_ fragment of the subject compound display large isotropic displacement parameters. Given the terminal nature of this fragment, significant thermal motion is likely the cause, although positional disorder cannot be excluded. In contrast, the analogous displacement parameters in the Wigley derivative appear regular, being comparable in size to other terminal carbon atoms in the main part of the structure.

The structure of (I) contains a weak C12—H12A···Cl1 interaction [H···Cl = 2.89 (2) Å, symmetry code: 3/2 - *x*, 1/2 + *y*, *z*; c.f. sum of van der Waals radii of H and Cl = 2.95 Å (Bondi, 1964)]. This links adjacent molecules forming chains which are almost parallel to the *y*-axis (see Figure 3). Adjacent chains are arranged anti-parallel to each other thus completing the three-dimensional structure. In contrast, no hydrogen-bonds or short non-bonded contacts are present in the diisopropylphenyl structure, as deduced from a search in Materials Mercury (Macrae *et al.*, 2008).

### Experimental

A solution of LiNH(2-*^t^*BuC_6_H_4_) (1.717 g, 11.07 mmol) in Et_2_O (80 ml) was added dropwise to a stirred solution of Cp*TaCl_4_ (1.267 g, 2.77 mmol) in Et_2_O (80 ml) at 0 °C. This mixture was allowed to warm up to room temperature and stirred for 24 h. The resultant yellow/brown solution was filtered from the white residue of LiCl, concentrated and cooled to -30 °C to yield long yellow crystals of (I) (yield: 1.47 g, 73%).

Elemental analysis for C_34_H_51_N_2_OClLiTa (727.14) found (required): %C = 56.17 (56.16), %H = 7.10 (7.07), %N = 3.82 (3.85).

Mass Spectrometry data (EI, m/*z*, ^35^Cl): 646 [*M* - LiOEt_2_]^+^.

^1^H NMR data (400 MHz, C_6_D_6_, 298 K): 0.57 (broad t, OCH_2_C*H_3_*), 1.62 (s, 18H, C*Me_3_*), 2.08 (s, 15H, C_5_*Me_5_*), 2.69 (broad q, OC*H_2_*CH_3_), 6.64 (d, ^2^H, *J* = 7.6 Hz, H^3^), 6.70, 7.05 (two t, 4H, *J* = 7.4 Hz, H^4^ and H^5^), 7.32 (d, 2H, *J* = 7.8 Hz, H^6^).

^13^C NMR data (100 MHz, C_6_D_6_, 298 K): 11.2 (q, *J* = 127 Hz, C_5_*Me_5_*), 14.4 (q, *J* = 127 Hz, OCH_2_*C*H_3_), 29.6 (q, *J* = 125 Hz, C*Me_3_*), 35.7 (s, *C*Me_3_), 64.6 (t, *J* = 143 Hz, O*C*H_2_CH_3_), 116.7 (s, *C_5_*Me_5_), 118.9, 125.3, 126.3, 126.9 (doublets, *J* = 154–159 Hz, C^3–6^), 140.5 (s, C^2^), 158.9 (s, C^1^).

### Refinement

A yellow rectangular crystal was mounted onto a Siemens *SMART*-CCD diffractometer using the oil-drop method (Kottke & Stalke, 1993).

Positional and anisotropic displacement parameters for all non-hydrogen atoms in the anionic part of the molecule were refined. Likewise, the lithium atom within the cation was refined anisotropically. The displacement parameters of the oxydiethyl group were refined isotropically. All hydrogen isotropic displacement parameters in the molecule were constrained to the riding model, *U*_iso_(H) = 1.2*U*_eq_(C) except for those relating to terminal methyl group H atoms, where *U*_iso_(H) = 1.5*U*_eq_(C).

### Crystal data

**Table:**

[LiTa(C_10_H_15_)(C_10_H_13_N)_2_Cl(C_4_H_10_O)]	*F*(000) = 2960
*M**_r_* = 727.11	*D*_x_ = 1.417 Mg m^−^^3^
Orthorhombic, *P**b**c**a*	Mo *K*α radiation, λ = 0.71073 Å
Hall symbol: -P 2ac 2ab	Cell parameters from 498 reflections
*a* = 19.5365 (12) Å	θ = 4.0–21.1°
*b* = 16.3544 (10) Å	µ = 3.33 mm^−^^1^
*c* = 21.3272 (13) Å	*T* = 150 K
*V* = 6814.2 (7) Å^3^	Rectangular block, yellow
*Z* = 8	0.60 × 0.34 × 0.16 mm

### Data collection

**Table:**

Siemens SMART CCD diffractometer	4848 independent reflections
Radiation source: fine-focus sealed tube	4792 reflections with *I* > 2σ(*I*)
graphite	*R*_int_ = 0.060
ω scans	θ_max_ = 23.3°, θ_min_ = 1.9°
Absorption correction: multi-scan (*SADABS*; Siemens, 1995)	*h* = −20→21
*T*_min_ = 0.346, *T*_max_ = 0.666	*k* = −17→18
24495 measured reflections	*l* = −23→21

### Refinement

**Table:**

Refinement on *F*^2^	Primary atom site location: heavy-atom method
Least-squares matrix: full	Secondary atom site location: difference Fourier map
*R*[*F*^2^ > 2σ(*F*^2^)] = 0.053	Hydrogen site location: inferred from neighbouring sites
*wR*(*F*^2^) = 0.105	H-atom parameters constrained
*S* = 1.23	*w* = 1/[σ^2^(*F*_o_^2^) + (0.0141*P*)^2^ + 61.7377*P*] where *P* = (*F*_o_^2^ + 2*F*_c_^2^)/3
4848 reflections	(Δ/σ)_max_ = 0.001
337 parameters	Δρ_max_ = 1.29 e Å^−^^3^
1 restraint	Δρ_min_ = −0.95 e Å^−^^3^

### Special details

**Table:**

Geometry. All e.s.d.'s (except the e.s.d. in the dihedral angle between two l.s. planes) are estimated using the full covariance matrix. The cell e.s.d.'s are taken into account individually in the estimation of e.s.d.'s in distances, angles and torsion angles; correlations between e.s.d.'s in cell parameters are only used when they are defined by crystal symmetry. An approximate (isotropic) treatment of cell e.s.d.'s is used for estimating e.s.d.'s involving l.s. planes.
Refinement. Refinement of *F*^2^ against ALL reflections. The weighted *R*-factor *wR* and goodness of fit *S* are based on *F*^2^, conventional *R*-factors *R* are based on *F*, with *F* set to zero for negative *F*^2^. The threshold expression of *F*^2^ > σ(*F*^2^) is used only for calculating *R*-factors(gt) *etc*. and is not relevant to the choice of reflections for refinement. *R*-factors based on *F*^2^ are statistically about twice as large as those based on *F*, and *R*- factors based on ALL data will be even larger.

### Fractional atomic coordinates and isotropic or equivalent isotropic displacement parameters (Å^2^)

**Table:**

	*x*	*y*	*z*	*U*_iso_*/*U*_eq_	
Ta1	0.770702 (15)	0.594882 (18)	−0.131442 (14)	0.02547 (13)	
Cl1	0.76902 (10)	0.45343 (11)	−0.10178 (10)	0.0383 (5)	
C1	0.8795 (4)	0.5664 (5)	−0.1864 (4)	0.0326 (18)	
C2	0.8751 (4)	0.6522 (5)	−0.1761 (4)	0.035 (2)	
C3	0.8178 (4)	0.6815 (4)	−0.2118 (4)	0.0323 (19)	
C4	0.7888 (4)	0.6145 (5)	−0.2446 (3)	0.0315 (18)	
C5	0.8262 (4)	0.5434 (5)	−0.2284 (3)	0.0294 (18)	
C6	0.9339 (4)	0.5112 (5)	−0.1595 (4)	0.043 (2)	
H6A	0.9639	0.5429	−0.1318	0.052*	
H6B	0.9610	0.4876	−0.1937	0.052*	
H6C	0.9122	0.4671	−0.1355	0.052*	
C7	0.9227 (5)	0.7023 (6)	−0.1368 (4)	0.046 (2)	
H7A	0.9574	0.6667	−0.1180	0.055*	
H7B	0.8968	0.7297	−0.1035	0.055*	
H7C	0.9452	0.7435	−0.1631	0.055*	
C8	0.7991 (5)	0.7695 (5)	−0.2201 (4)	0.045 (2)	
H8A	0.8268	0.8032	−0.1919	0.054*	
H8B	0.7505	0.7771	−0.2102	0.054*	
H8C	0.8076	0.7859	−0.2636	0.054*	
C9	0.7304 (4)	0.6190 (5)	−0.2893 (4)	0.043 (2)	
H9A	0.7203	0.5642	−0.3053	0.051*	
H9B	0.7426	0.6550	−0.3243	0.051*	
H9C	0.6901	0.6408	−0.2677	0.051*	
C10	0.8139 (5)	0.4592 (5)	−0.2540 (4)	0.040 (2)	
H10A	0.7740	0.4602	−0.2819	0.048*	
H10B	0.8054	0.4212	−0.2194	0.048*	
H10C	0.8542	0.4412	−0.2776	0.048*	
N1	0.7902 (3)	0.6469 (4)	−0.0570 (3)	0.0278 (14)	
C11	0.8143 (4)	0.6997 (4)	−0.0099 (4)	0.0283 (17)	
C12	0.8098 (4)	0.7845 (5)	−0.0219 (4)	0.0342 (19)	
H12A	0.7937	0.8021	−0.0617	0.041*	
C13	0.8277 (5)	0.8422 (5)	0.0214 (4)	0.045 (2)	
H13A	0.8236	0.8988	0.0121	0.054*	
C14	0.8519 (5)	0.8165 (5)	0.0790 (4)	0.048 (2)	
H14A	0.8637	0.8556	0.1101	0.057*	
C15	0.8590 (5)	0.7337 (5)	0.0915 (4)	0.044 (2)	
H15A	0.8765	0.7175	0.1311	0.053*	
C16	0.8415 (4)	0.6732 (5)	0.0483 (3)	0.0285 (17)	
C17	0.8502 (4)	0.5813 (5)	0.0649 (4)	0.0356 (19)	
C18	0.7817 (5)	0.5367 (5)	0.0635 (4)	0.048 (2)	
H18A	0.7887	0.4789	0.0739	0.058*	
H18B	0.7617	0.5410	0.0215	0.058*	
H18C	0.7506	0.5614	0.0942	0.058*	
C19	0.9004 (5)	0.5425 (6)	0.0171 (5)	0.051 (2)	
H19A	0.9064	0.4844	0.0269	0.061*	
H19B	0.9447	0.5704	0.0196	0.061*	
H19C	0.8818	0.5482	−0.0253	0.061*	
C20	0.8814 (5)	0.5694 (6)	0.1295 (4)	0.053 (3)	
H20A	0.8863	0.5109	0.1381	0.064*	
H20B	0.8516	0.5943	0.1612	0.064*	
H20C	0.9266	0.5955	0.1310	0.064*	
N2	0.6791 (3)	0.6225 (4)	−0.1398 (3)	0.0286 (14)	
C21	0.6174 (4)	0.6516 (5)	−0.1624 (3)	0.0291 (17)	
C22	0.6147 (4)	0.7345 (5)	−0.1801 (4)	0.039 (2)	
H22A	0.6547	0.7670	−0.1757	0.047*	
C23	0.5558 (5)	0.7708 (5)	−0.2039 (4)	0.044 (2)	
H23A	0.5560	0.8267	−0.2161	0.052*	
C24	0.4974 (5)	0.7246 (6)	−0.2094 (4)	0.042 (2)	
H24A	0.4567	0.7481	−0.2259	0.051*	
C25	0.4985 (4)	0.6440 (5)	−0.1908 (4)	0.037 (2)	
H25A	0.4575	0.6131	−0.1947	0.044*	
C26	0.5559 (4)	0.6054 (5)	−0.1667 (3)	0.0301 (18)	
C27	0.5531 (4)	0.5142 (5)	−0.1470 (4)	0.037 (2)	
C28	0.4805 (5)	0.4798 (6)	−0.1530 (5)	0.055 (3)	
H28A	0.4497	0.5102	−0.1252	0.066*	
H28B	0.4805	0.4219	−0.1411	0.066*	
H28C	0.4649	0.4853	−0.1965	0.066*	
C29	0.5753 (5)	0.5038 (5)	−0.0792 (4)	0.046 (2)	
H29A	0.6217	0.5256	−0.0739	0.055*	
H29B	0.5748	0.4456	−0.0682	0.055*	
H29C	0.5437	0.5336	−0.0517	0.055*	
C30	0.5987 (5)	0.4636 (5)	−0.1916 (5)	0.049 (2)	
H30A	0.6460	0.4836	−0.1892	0.059*	
H30B	0.5819	0.4693	−0.2347	0.059*	
H30C	0.5973	0.4059	−0.1792	0.059*	
Li1	0.6892 (8)	0.6800 (10)	−0.0542 (7)	0.049 (4)	
O1	0.6296 (6)	0.7269 (7)	0.0070 (6)	0.124 (4)*	
C50	0.6498 (19)	0.759 (2)	0.0679 (18)	0.256 (15)*	
H50A	0.6991	0.7731	0.0673	0.308*	
H50B	0.6237	0.8095	0.0770	0.308*	
C51	0.6380 (13)	0.7041 (15)	0.1120 (12)	0.183 (10)*	
H51A	0.6590	0.7220	0.1514	0.274*	
H51B	0.6576	0.6515	0.0994	0.274*	
H51C	0.5885	0.6980	0.1180	0.274*	
C61	0.5323 (12)	0.7245 (14)	−0.0022 (11)	0.179 (9)*	
H61A	0.4853	0.7428	−0.0098	0.269*	
H61B	0.5371	0.7075	0.0417	0.269*	
H61C	0.5428	0.6782	−0.0297	0.269*	
C60	0.5824 (19)	0.795 (2)	−0.016 (2)	0.33 (2)*	
H60A	0.5873	0.8105	−0.0605	0.398*	
H60B	0.5785	0.8432	0.0122	0.398*	

### Atomic displacement parameters (Å^2^)

**Table:**

	*U*^11^	*U*^22^	*U*^33^	*U*^12^	*U*^13^	*U*^23^
Ta1	0.02539 (19)	0.02426 (18)	0.02676 (19)	−0.00208 (13)	−0.00071 (13)	0.00144 (13)
Cl1	0.0452 (12)	0.0255 (10)	0.0441 (11)	0.0005 (9)	0.0013 (10)	0.0065 (9)
C1	0.027 (4)	0.040 (5)	0.031 (4)	−0.003 (4)	0.004 (4)	−0.005 (4)
C2	0.036 (5)	0.041 (5)	0.029 (4)	−0.014 (4)	0.012 (4)	−0.004 (4)
C3	0.041 (5)	0.022 (4)	0.034 (4)	−0.009 (4)	0.011 (4)	0.002 (3)
C4	0.037 (5)	0.036 (4)	0.022 (4)	−0.006 (4)	0.003 (3)	0.002 (3)
C5	0.028 (4)	0.034 (4)	0.026 (4)	−0.004 (3)	0.005 (3)	−0.005 (3)
C6	0.030 (5)	0.050 (5)	0.050 (5)	0.005 (4)	0.006 (4)	−0.005 (4)
C7	0.037 (5)	0.052 (6)	0.048 (5)	−0.015 (4)	0.014 (4)	−0.012 (4)
C8	0.059 (6)	0.030 (4)	0.046 (5)	−0.009 (4)	0.016 (5)	0.005 (4)
C9	0.049 (6)	0.041 (5)	0.038 (5)	0.000 (4)	−0.007 (4)	−0.002 (4)
C10	0.040 (5)	0.036 (4)	0.045 (5)	0.003 (4)	0.003 (4)	−0.009 (4)
N1	0.026 (3)	0.025 (3)	0.032 (4)	0.000 (3)	0.005 (3)	0.005 (3)
C11	0.029 (4)	0.023 (4)	0.033 (4)	0.001 (3)	0.003 (4)	0.001 (3)
C12	0.045 (5)	0.030 (4)	0.027 (4)	0.000 (4)	−0.001 (4)	0.001 (3)
C13	0.059 (6)	0.033 (5)	0.042 (5)	−0.004 (4)	−0.006 (5)	−0.003 (4)
C14	0.053 (6)	0.042 (5)	0.047 (6)	−0.005 (4)	−0.001 (5)	−0.017 (4)
C15	0.047 (5)	0.047 (5)	0.039 (5)	0.004 (4)	−0.011 (4)	−0.001 (4)
C16	0.024 (4)	0.038 (4)	0.024 (4)	0.001 (3)	−0.004 (3)	0.001 (3)
C17	0.042 (5)	0.037 (5)	0.027 (4)	0.012 (4)	−0.010 (4)	0.002 (4)
C18	0.066 (6)	0.041 (5)	0.038 (5)	−0.005 (5)	−0.007 (5)	0.011 (4)
C19	0.049 (6)	0.042 (5)	0.062 (6)	0.015 (5)	−0.008 (5)	0.005 (5)
C20	0.062 (6)	0.055 (6)	0.042 (5)	0.013 (5)	−0.016 (5)	0.006 (4)
N2	0.029 (4)	0.020 (3)	0.037 (4)	0.000 (3)	−0.005 (3)	0.005 (3)
C21	0.024 (4)	0.037 (4)	0.027 (4)	0.001 (3)	−0.002 (3)	−0.001 (3)
C22	0.034 (5)	0.032 (5)	0.052 (5)	−0.003 (4)	−0.006 (4)	−0.001 (4)
C23	0.051 (6)	0.034 (5)	0.047 (5)	0.011 (4)	−0.003 (4)	0.004 (4)
C24	0.035 (5)	0.054 (6)	0.039 (5)	0.012 (4)	−0.010 (4)	−0.002 (4)
C25	0.026 (4)	0.047 (5)	0.037 (5)	−0.001 (4)	−0.001 (4)	−0.009 (4)
C26	0.028 (4)	0.037 (5)	0.025 (4)	0.000 (4)	0.004 (3)	−0.004 (3)
C27	0.032 (5)	0.034 (5)	0.045 (5)	−0.009 (4)	0.001 (4)	0.005 (4)
C28	0.040 (5)	0.046 (6)	0.079 (7)	−0.011 (4)	0.000 (5)	0.004 (5)
C29	0.045 (5)	0.042 (5)	0.051 (6)	−0.004 (4)	0.010 (5)	0.008 (4)
C30	0.045 (6)	0.038 (5)	0.063 (6)	−0.001 (4)	0.000 (5)	−0.007 (4)
Li1	0.035 (8)	0.069 (10)	0.045 (9)	0.012 (7)	0.000 (7)	−0.011 (8)

### Geometric parameters (Å, °)

**Table:**

Ta1—N1	1.842 (6)	C17—C18	1.526 (12)
Ta1—N2	1.854 (6)	C17—C19	1.549 (12)
Ta1—Cl1	2.3985 (19)	C18—H18A	0.9800
Ta1—C3	2.405 (7)	C18—H18B	0.9800
Ta1—C2	2.438 (8)	C18—H18C	0.9800
Ta1—C4	2.460 (7)	C19—H19A	0.9800
Ta1—C1	2.472 (8)	C19—H19B	0.9800
Ta1—C5	2.481 (7)	C19—H19C	0.9800
Ta1—Li1	2.681 (15)	C20—H20A	0.9800
C1—C2	1.422 (11)	C20—H20B	0.9800
C1—C5	1.423 (11)	C20—H20C	0.9800
C1—C6	1.507 (11)	N2—C21	1.383 (10)
C2—C3	1.435 (12)	C21—C22	1.409 (11)
C2—C7	1.499 (11)	C21—C26	1.422 (10)
C3—C4	1.417 (11)	C21—Li1	2.740 (17)
C3—C8	1.496 (11)	C22—C23	1.389 (12)
C4—C5	1.416 (11)	C22—H22A	0.9500
C4—C9	1.488 (11)	C23—C24	1.374 (12)
C5—C10	1.502 (11)	C23—H23A	0.9500
C6—H6A	0.9800	C24—C25	1.378 (12)
C6—H6B	0.9800	C24—H24A	0.9500
C6—H6C	0.9800	C25—C26	1.386 (11)
C7—H7A	0.9800	C25—H25A	0.9500
C7—H7B	0.9800	C26—C27	1.551 (11)
C7—H7C	0.9800	C27—C29	1.521 (12)
C8—H8A	0.9800	C27—C28	1.531 (12)
C8—H8B	0.9800	C27—C30	1.543 (12)
C8—H8C	0.9800	C28—H28A	0.9800
C9—H9A	0.9800	C28—H28B	0.9800
C9—H9B	0.9800	C28—H28C	0.9800
C9—H9C	0.9800	C29—H29A	0.9800
C10—H10A	0.9800	C29—H29B	0.9800
C10—H10B	0.9800	C29—H29C	0.9800
C10—H10C	0.9800	C30—H30A	0.9800
N1—C11	1.405 (10)	C30—H30B	0.9800
Li1—N1	2.048 (16)	C30—H30C	0.9800
Li1—N2	2.062 (16)	O1—C50	1.46 (3)
Li1—O1	1.910 (19)	O1—C60	1.527 (18)
C11—C12	1.412 (10)	C50—C51	1.32 (3)
C11—C16	1.418 (10)	C50—H50A	0.9900
C11—Li1	2.641 (17)	C50—H50B	0.9900
C12—C13	1.366 (11)	C51—H51A	0.9800
C12—H12A	0.9500	C51—H51B	0.9800
C13—C14	1.382 (13)	C51—H51C	0.9800
C13—H13A	0.9500	C61—C60	1.54 (4)
C14—C15	1.387 (12)	C61—H61A	0.9800
C14—H14A	0.9500	C61—H61B	0.9800
C15—C16	1.394 (11)	C61—H61C	0.9800
C15—H15A	0.9500	C60—H60A	0.9900
C16—C17	1.554 (11)	C60—H60B	0.9900
C17—C20	1.521 (11)		
			
N1—Ta1—N2	99.8 (3)	C15—C16—C11	117.0 (7)
N1—Ta1—Cl1	102.76 (19)	C15—C16—C17	120.6 (7)
N2—Ta1—Cl1	104.27 (18)	C11—C16—C17	122.4 (7)
N1—Ta1—C3	105.3 (3)	C20—C17—C18	108.0 (7)
N2—Ta1—C3	99.1 (3)	C20—C17—C19	106.9 (7)
Cl1—Ta1—C3	139.54 (19)	C18—C17—C19	110.3 (7)
N1—Ta1—C2	89.2 (3)	C20—C17—C16	112.0 (7)
N2—Ta1—C2	132.6 (3)	C18—C17—C16	111.2 (7)
Cl1—Ta1—C2	119.0 (2)	C19—C17—C16	108.4 (7)
C3—Ta1—C2	34.5 (3)	C17—C18—H18A	109.5
N1—Ta1—C4	139.1 (3)	C17—C18—H18B	109.5
N2—Ta1—C4	90.8 (3)	H18A—C18—H18B	109.5
Cl1—Ta1—C4	112.73 (18)	C17—C18—H18C	109.5
C3—Ta1—C4	33.9 (3)	H18A—C18—H18C	109.5
C2—Ta1—C4	56.4 (3)	H18B—C18—H18C	109.5
N1—Ta1—C1	108.5 (3)	C17—C19—H19A	109.5
N2—Ta1—C1	146.2 (3)	C17—C19—H19B	109.5
Cl1—Ta1—C1	87.41 (19)	H19A—C19—H19B	109.5
C3—Ta1—C1	56.3 (3)	C17—C19—H19C	109.5
C2—Ta1—C1	33.7 (3)	H19A—C19—H19C	109.5
C4—Ta1—C1	55.7 (3)	H19B—C19—H19C	109.5
N1—Ta1—C5	141.7 (3)	C17—C20—H20A	109.5
N2—Ta1—C5	115.1 (3)	C17—C20—H20B	109.5
Cl1—Ta1—C5	84.18 (19)	H20A—C20—H20B	109.5
C3—Ta1—C5	55.9 (3)	C17—C20—H20C	109.5
C2—Ta1—C5	55.9 (3)	H20A—C20—H20C	109.5
C4—Ta1—C5	33.3 (3)	H20B—C20—H20C	109.5
C1—Ta1—C5	33.4 (2)	C21—N2—Ta1	163.4 (5)
N1—Ta1—Li1	49.7 (4)	C21—N2—Li1	103.6 (6)
N2—Ta1—Li1	50.1 (4)	Ta1—N2—Li1	86.2 (5)
Cl1—Ta1—Li1	109.3 (4)	N2—C21—C22	117.3 (7)
C3—Ta1—Li1	111.0 (4)	N2—C21—C26	125.1 (7)
C2—Ta1—Li1	122.5 (4)	C22—C21—C26	117.5 (7)
C4—Ta1—Li1	128.3 (4)	N2—C21—Li1	47.0 (5)
C1—Ta1—Li1	154.2 (4)	C22—C21—Li1	94.7 (6)
C5—Ta1—Li1	161.4 (4)	C26—C21—Li1	125.2 (6)
C2—C1—C5	108.1 (7)	C23—C22—C21	122.7 (8)
C2—C1—C6	125.2 (7)	C23—C22—H22A	118.7
C5—C1—C6	126.6 (7)	C21—C22—H22A	118.7
C2—C1—Ta1	71.9 (4)	C24—C23—C22	119.0 (8)
C5—C1—Ta1	73.7 (4)	C24—C23—H23A	120.5
C6—C1—Ta1	122.6 (5)	C22—C23—H23A	120.5
C1—C2—C3	107.2 (7)	C23—C24—C25	119.2 (8)
C1—C2—C7	126.0 (8)	C23—C24—H24A	120.4
C3—C2—C7	126.8 (8)	C25—C24—H24A	120.4
C1—C2—Ta1	74.5 (4)	C24—C25—C26	123.7 (8)
C3—C2—Ta1	71.5 (4)	C24—C25—H25A	118.1
C7—C2—Ta1	120.7 (5)	C26—C25—H25A	118.1
C4—C3—C2	108.4 (7)	C25—C26—C21	117.8 (7)
C4—C3—C8	126.0 (8)	C25—C26—C27	120.6 (7)
C2—C3—C8	125.1 (7)	C21—C26—C27	121.6 (7)
C4—C3—Ta1	75.2 (4)	C29—C27—C28	107.6 (7)
C2—C3—Ta1	74.0 (4)	C29—C27—C30	111.2 (7)
C8—C3—Ta1	123.9 (6)	C28—C27—C30	106.7 (7)
C5—C4—C3	107.9 (7)	C29—C27—C26	110.8 (7)
C5—C4—C9	126.4 (7)	C28—C27—C26	111.3 (7)
C3—C4—C9	125.7 (7)	C30—C27—C26	109.2 (7)
C5—C4—Ta1	74.2 (4)	C27—C28—H28A	109.5
C3—C4—Ta1	71.0 (4)	C27—C28—H28B	109.5
C9—C4—Ta1	121.7 (5)	H28A—C28—H28B	109.5
C4—C5—C1	108.4 (7)	C27—C28—H28C	109.5
C4—C5—C10	125.5 (7)	H28A—C28—H28C	109.5
C1—C5—C10	126.1 (7)	H28B—C28—H28C	109.5
C4—C5—Ta1	72.5 (4)	C27—C29—H29A	109.5
C1—C5—Ta1	72.9 (4)	C27—C29—H29B	109.5
C10—C5—Ta1	123.0 (5)	H29A—C29—H29B	109.5
C1—C6—H6A	109.5	C27—C29—H29C	109.5
C1—C6—H6B	109.5	H29A—C29—H29C	109.5
H6A—C6—H6B	109.5	H29B—C29—H29C	109.5
C1—C6—H6C	109.5	C27—C30—H30A	109.5
H6A—C6—H6C	109.5	C27—C30—H30B	109.5
H6B—C6—H6C	109.5	H30A—C30—H30B	109.5
C2—C7—H7A	109.5	C27—C30—H30C	109.5
C2—C7—H7B	109.5	H30A—C30—H30C	109.5
H7A—C7—H7B	109.5	H30B—C30—H30C	109.5
C2—C7—H7C	109.5	O1—Li1—N1	135.5 (9)
H7A—C7—H7C	109.5	O1—Li1—N2	136.9 (9)
H7B—C7—H7C	109.5	N1—Li1—N2	86.9 (6)
C3—C8—H8A	109.5	O1—Li1—C11	105.7 (8)
C3—C8—H8B	109.5	N1—Li1—C11	31.8 (3)
H8A—C8—H8B	109.5	N2—Li1—C11	117.4 (7)
C3—C8—H8C	109.5	O1—Li1—Ta1	172.1 (10)
H8A—C8—H8C	109.5	N1—Li1—Ta1	43.3 (3)
H8B—C8—H8C	109.5	N2—Li1—Ta1	43.6 (3)
C4—C9—H9A	109.5	C11—Li1—Ta1	74.5 (4)
C4—C9—H9B	109.5	O1—Li1—C21	109.4 (7)
H9A—C9—H9B	109.5	N1—Li1—C21	115.1 (7)
C4—C9—H9C	109.5	N2—Li1—C21	29.4 (3)
H9A—C9—H9C	109.5	C11—Li1—C21	142.7 (6)
H9B—C9—H9C	109.5	Ta1—Li1—C21	72.5 (4)
C5—C10—H10A	109.5	C50—O1—C60	101 (2)
C5—C10—H10B	109.5	C50—O1—Li1	126.2 (18)
H10A—C10—H10B	109.5	C60—O1—Li1	116.3 (19)
C5—C10—H10C	109.5	C51—C50—O1	110 (3)
H10A—C10—H10C	109.5	C51—C50—H50A	109.7
H10B—C10—H10C	109.5	O1—C50—H50A	109.7
C11—N1—Ta1	165.7 (5)	C51—C50—H50B	109.7
C11—N1—Li1	98.1 (6)	O1—C50—H50B	109.7
Ta1—N1—Li1	87.0 (5)	H50A—C50—H50B	108.2
N1—C11—C12	116.9 (7)	C50—C51—H51A	109.5
N1—C11—C16	124.3 (6)	C50—C51—H51B	109.5
C12—C11—C16	118.8 (7)	H51A—C51—H51B	109.5
N1—C11—Li1	50.1 (5)	C50—C51—H51C	109.5
C12—C11—Li1	89.8 (6)	H51A—C51—H51C	109.5
C16—C11—Li1	128.5 (6)	H51B—C51—H51C	109.5
C13—C12—C11	122.6 (8)	C60—C61—H61A	109.5
C13—C12—H12A	118.7	C60—C61—H61B	109.5
C11—C12—H12A	118.7	H61A—C61—H61B	109.5
C12—C13—C14	118.6 (8)	C60—C61—H61C	109.5
C12—C13—H13A	120.7	H61A—C61—H61C	109.5
C14—C13—H13A	120.7	H61B—C61—H61C	109.5
C13—C14—C15	120.1 (8)	O1—C60—C61	77.0 (18)
C13—C14—H14A	120.0	O1—C60—H60A	115.7
C15—C14—H14A	120.0	C61—C60—H60A	115.7
C14—C15—C16	122.8 (8)	O1—C60—H60B	115.7
C14—C15—H15A	118.6	C61—C60—H60B	115.7
C16—C15—H15A	118.6	H60A—C60—H60B	112.7

### Hydrogen-bond geometry (Å, °)

**Table:**

*D*—H···*A*	*D*—H	H···*A*	*D*···*A*	*D*—H···*A*
C12—H12A···Cl1^i^	0.95	2.89	3.593 (8)	132

Symmetry codes: (i) −*x*+3/2, *y*+1/2, *z*.
